# Comparisons of Retinal Nerve Fiber Layer Thickness after Indocyanine Green, Brilliant Blue G, or Triamcinolone Acetonide-Assisted Macular Hole Surgery

**DOI:** 10.1155/2014/187308

**Published:** 2014-05-19

**Authors:** Yoshiharu Toba, Shigeki Machida, Daijiro Kurosaka

**Affiliations:** ^1^Department of Ophthalmology, Iwate Medical University, School of Medicine, 19-1 Uchimaru, Morioka, Iwate 020-8505, Japan; ^2^Department of Ophthalmology, Dokkyo Medical University Koshigaya Hospital, 2-1-50 Minami-Koshigaya, Koshigaya, Saitama 343-8555, Japan

## Abstract

*Purpose*. To compare the postoperative changes of the retinal nerve fiber layer (RNFL) thickness in patients with macular holes (MHs) treated with vitrectomy with indocyanine green- (ICG-), brilliant blue G- (BBG-), or triamcinolone acetonide- (TA-)assisted internal limiting membrane (ILM) peeling. *Methods*. Sixty-one eyes of 61 consecutive patients with MHs were studied. Each eye was randomly selected to undergo either ICG- (*n* = 18), BBG- (*n* = 21), or TA-assisted (*n* = 22) ILM peeling. The circumferential retinal nerve fiber layer (RNFL) thickness was determined by spectral-domain optical coherence tomography (SD-OCT) before and 1, 3, 6, 9, and 12 months postoperatively. The mean overall and the sectoral thicknesses of the RNFL were obtained for each group. *Results*. A transient increase of the RNFL thickness was seen in the mean overall and sectoral thicknesses except for the nasal/inferior sector at 1 month after surgery for the three groups. Then, the thickness gradually decreased and returned to the baseline level in all sectors except for the nasal/inferior sector. The differences in the RNFL thickness among the groups were not significant for at least 12 months postoperatively. *Conclusions*. The degree of change of the RNFL thickness was not significantly related to the type of vital stain used during MH surgery.

## 1. Introduction

Peeling the internal limiting membrane (ILM) during macular hole (MH) surgery has become an important procedure that improves the anatomical success and decreases the recurrence rates [1–3]. Several vital dyes have been used to make the ILM more visible which facilitated the surgical procedures. The first dye used for MH surgery was indocyanine green [4, 5]; however ICG was shown to be toxic to the retina at the concentrations used during vitrectomy [6, 7]. Thus, exposure of retinal ganglion cells (RGCs) to ICG* in vitro* and* in vivo* was shown to damage the RGCs [8, 9]. In addition, visual field defects have been reported after using ICG during vitrectomy [10–13].

Brilliant blue G (BBG) emerged as an alternative dye which selectively stains the ILM. Laboratory studies on rats and monkeys demonstrated that BBG was less toxic to the retina than ICG [14–16], although high concentrations or long exposures to BBG can damage the RGCs [9].

Triamcinolone acetonide (TA) is a steroidal compound that has also been used to make the posterior vitreous membrane [17, 18] and ILM more visible [19–21]. TA is not soluble in the vitreous, and the presence of TA particles on the retinal surface enables surgeons to see where the ILM has been peeled as the area lacking white specks.

We recently compared the function of RGCs using the photopic negative response (PhNR) [22] of the cone electroretinogram in patients who had undergone indocyanine ICG-, BBG-, or TA-assisted ILM peeling during MH surgery [23]. We found that TA- and BBG-assisted ILM peeling resulted in less decrease in the PhNR amplitude than after ICG-assisted vitrectomy. In the MH patients treated with vitrectomy with ICG-assisted ILM peeling, the PhNR amplitude was significantly decreased at 1 month after the surgery and then gradually recovered. However, it did not return to the baseline level even at 12 months after the surgery suggesting a permanent loss of RGC function. On the other hand, in patients who underwent BBG- or TA-assisted ILM peeling, the PhNR amplitude returned to the baseline level as early as 1 month after surgery. These results motivated us to examine the morphometric parameters, for example, the retinal nerve fiber layer (RNFL) thickness, of the RGCs after MH surgery.

The RNFL thickness around the optic nerve head has been measured by optical coherence tomography (OCT) and used to evaluate the intactness of the RGC axons in eyes with glaucoma and optic nerve diseases [24]. Recently, a transient increase of the RNFL thickness after ILM peeling has been reported by two groups of investigators [25, 26], but another group of investigators has reported a reduction of RNFL thickness in some sectors after vitrectomy [27]. A thinning of the RNFL and visual field defects were also reported in MH patients who had undergone ICG-assisted ILM peeling [12]. This raised the possibility that the vital dyes could induce these changes of the RNFL thickness after MH surgery.

Thus, the purpose of the study was to compare the changes of the RNFL thickness as a function of postoperative periods in MH patients treated with vitrectomy with ICG-, BBG-, or TA-assisted ILM peeling.

## 2. Methods

### 2.1. Patients

All patients had a comprehensive ophthalmological examination including measurements of the best-corrected visual acuity (BCVA) with a Snellen chart, slit-lamp biomicroscopy, and indirect ophthalmoscopy. Spectral-domain OCT (SD-OCT) was used for staging the MH and confirming the postoperative closure of the MH.

We examined 61 eyes of 61 consecutive patients that had undergone vitrectomy with ILM peeling during MH surgery in our hospital from January 2011 to September 2012. All patients did not have any ocular disease other than a MH and cataract. The patients consisted of 38 women and 23 men whose mean age was 65.2 ± 7.6 (average ± standard deviation) years with a range from 47 to 80 years. Because nuclear cataracts commonly develop after vitrectomy in patients older than 50 years [28], all patients underwent vitrectomy combined with phacoemulsification and aspiration (PEA) with implantation of an intraocular lens (NX-70, Advanced Vision Science, Inc., Coleta, CA, USA).

### 2.2. Surgical Procedures

Each patient was randomly assigned to receive either IGG (*n* = 18), BBG (*n* = 21), or TA (*n* = 22). Preservative-free TA (MaQaid, Wakamoto Pharmaceutical Co, Ltd, Tokyo, Japan) was suspended in 4 mL balanced salt solution and injected intravitreally during vitrectomy to make the posterior hyaloid membrane more visible. For the ICG group, 25 milligrams of ICG (Diagnogreen, Daiichi Pharmaceutical Co., Ltd., Tokyo, Japan) was dissolved in 1 mL of sterilized distilled water and then diluted with 9 mL of balanced salt solution (BSS plus, Alcon Japan, Tokyo, Japan) to a final concentration of 2.5 mg/mL (0.25%). BBG was dissolved in BSS to a concentration of 0.025%. These dyes were drawn into a 1.0 mL syringe through a sterilized filter (Millex GS filter unit 0.22 *μ*m, Millipore Ireland Ltd, Cork, Ireland). Approximately 0.2 mL of the dye solutions was injected intravitreally with a gentle stream directed toward the posterior pole of the eye after removal of the posterior hyaloid membrane. The dye was removed from the vitreous cavity by infusion and aspiration as rapidly as possible.

The ILM was grasped with ILM forceps and peeled off the retina. Air-fluid exchange was performed followed by an injection of 20% sulfur hexafluoride (SF_6_). All surgical procedures were performed by a single surgeon (SM).

This research was conducted in accordance with the Institutional Guidelines of Iwate Medical University, and the procedures conformed to the tenets of the Declaration of Helsinki. An informed consent was obtained from all subjects after a full explanation of the nature of the experiments.

### 2.3. Optical Coherence Tomography (OCT)

**Figure 1:**
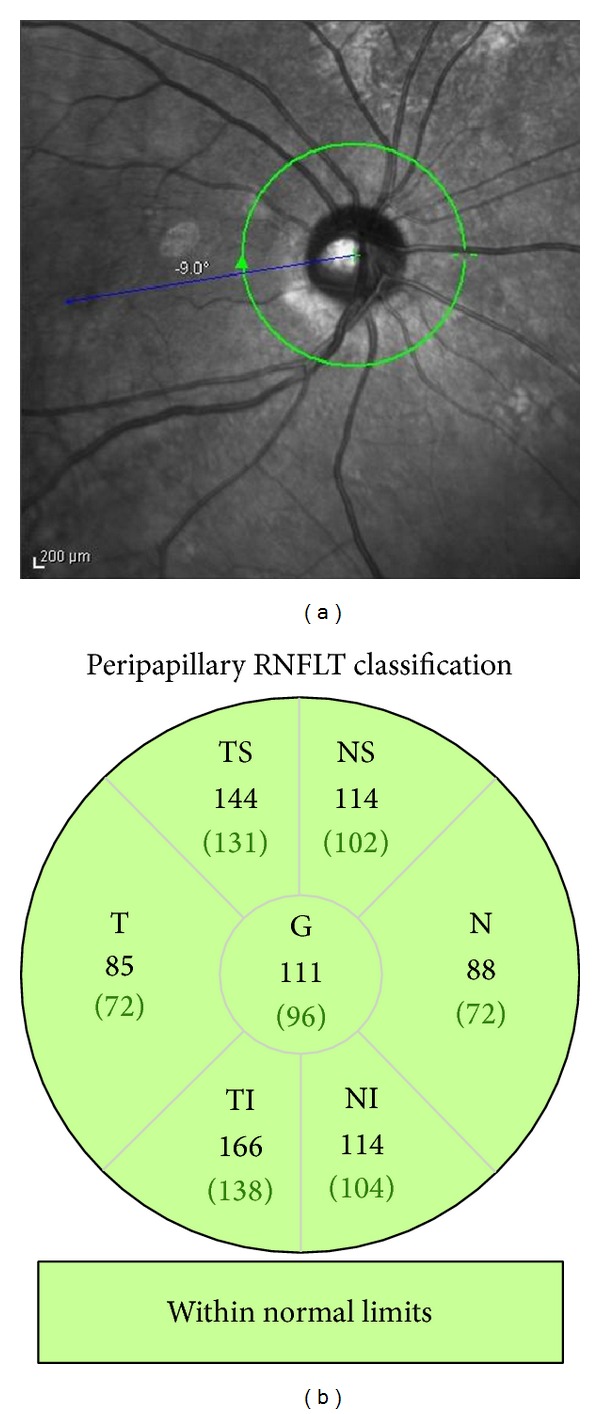
Global and sectoral retinal nerve fiber (RNFL) thicknesses before and after vitrectomy with ILM peeling for eyes with a macular hole (MH). (a) Peripapillary RNFL thickness was measured around the optic disc. (b) Sectoral analyses of the RNFL thickness were performed for the temporal/superior (TS, 1–45 degree), temporal (T, 46–135 degree), temporal/inferior (TI, 136–180 degree), nasal/inferior (NI, 181–225 degree), nasal (N, 226–315), and nasal/superior (NS, 316–360) sectors.

A closure of the macular hole was confirmed postoperatively in the SD-OCT images in all patients. Six, 9 mm radial scans passing through the fovea were performed at every visit using spectral-domain OCT (SD-OCT, Spectralis, Germany). To measure the thickness of the RNFL around the optic nerve head, circular scans of 1.8 mm radius were performed preoperatively and 1, 3, 6, 9, and 12 months after the surgery (Figure 1(a)). Each OCT image was obtained along a 360 degree path around the optic disc. The mean RNFLT thickness of the 1,536 measured points was called the global thickness. The averaged RNFL thickness of the temporal/superior (1° to 45°), temporal (46° to 135°), temporal/inferior (136° to 180°), nasal/inferior (181° to 225° degree), nasal (226° to 315°), and nasal/superior (316° to 360°) sectors was obtained for the sector analyses (Figure 1(b)).

### 2.4. Statistical Analyses

One-way repeated measures ANOVA was used to determine the statistical significance of the RNFL changes with postoperative time. In addition, Bonferroni's multiple comparison tests were performed after the ANOVA as post hoc tests.

Two-way repeated measures ANOVA was used to compare data among groups, and the Bonferroni post hoc test was performed following the ANOVA to determine the statistical significance between paired data at each time point. These analyses were performed using Prism 5.1 (GraphPad Software Inc. San Diego CA). The level of statistical significance was set at *P* < 0.05.

## 3. Results

### 3.1. Comparison between Groups

**Figure 2:**
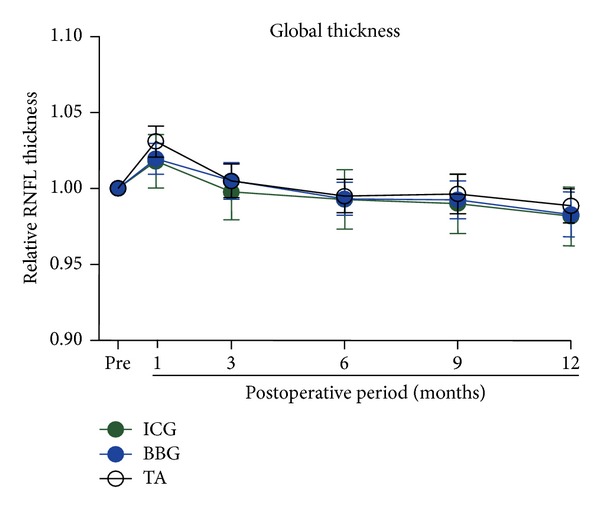
Means of the relative global RNFL thickness are plotted against the postoperative time for ICG, BBG, and TA groups. The RNFL thickness is expressed relative to the preoperative value. Green, blue, and black symbols represent ICG, BBG, and TA groups, respectively. The means ± SEMs. RNFL: retinal nerve fiber layer, ICG: indocyanine green, BBG: brilliant blue G, TA: triamcinolone acetonide.

The mean global thickness of the RNFL for ICG (green symbols), BBG (blue symbols), and TA groups (black symbols) is plotted as a function of postoperative periods in Figure 2. The RNFL thickness was normalized to the baseline value, and the values plotted are the relative RNFL thickness. Because the RNFL thickness has considerable variations among individuals, we used the relative thickness to compare the RNFL thickness among the groups. In all groups, the RNFL thickness was significantly increased at 1 month postoperatively followed by gradual decrease to the baseline level at 6 months after the surgery. The lines for each of the groups are overlapped indicating no significant difference in the changes of the global thickness of the RNFL among the three groups. In addition, the RNFL thickness at 12 months did not differ significantly from the baseline thickness.

**Figure 3:**
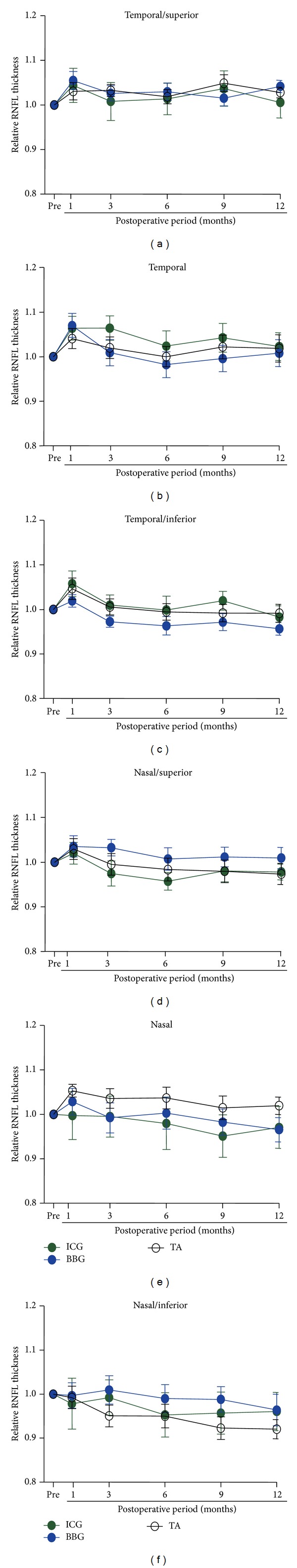
Means of the relative RNFL thickness are plotted as a function of postoperative periods for the temporal/superior (a), temporal (b), temporal/inferior (c), nasal/superior (d), nasal (e), and nasal/inferior (f) sector analyses. The RNFL thickness is expressed relative to the preoperative value. Green, blue, and black symbols represent ICG, BBG, and TA groups, respectively. Means ± SEMs. RNFL: retinal nerve fiber layer, ICG: indocyanine green, BBG: brilliant blue G, TA: triamcinolone acetonide.

The results of the sectoral analyses of the relative RNFL thickness are shown in Figure 3. A transient increase of the RNFL followed by a gradual reduction was seen in all sectors (Figures 3(a)–3(e)) expect for the nasal/inferior sector where the RNFL thickness continued to decrease up to 12 months (Figure 3(f)). The degree of decrease of the sector thickness was not significantly different among the three groups.

### 3.2. Combined Data of Three Groups

**Figure 4:**
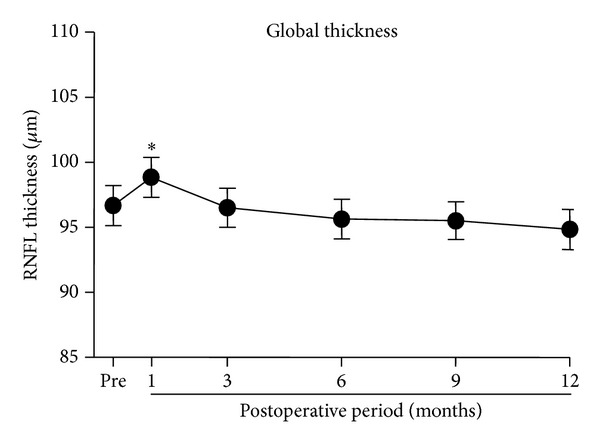
The means of the absolute global RNFL thickness are plotted against the postoperative time. Means ± SEMs. RNFL: retinal nerve fiber layer, *P < 0.01.

Because the global and sectoral analyses demonstrated that there was no significant difference in the RNFL thickness among the three groups, we combined the data from the three groups for further analyses using the absolute values of the RNFL thickness. The averages of the absolute values of the global RNFL thickness are plotted against pre- and postoperative times in Figure 4. The global RNFL thickness was significantly increased at one month and then slowly decreased thereafter (*P* < 0.0001). Multiple comparison tests showed that the increase in the thickness at 1 month was significant (*P* < 0.01).

**Figure 5:**
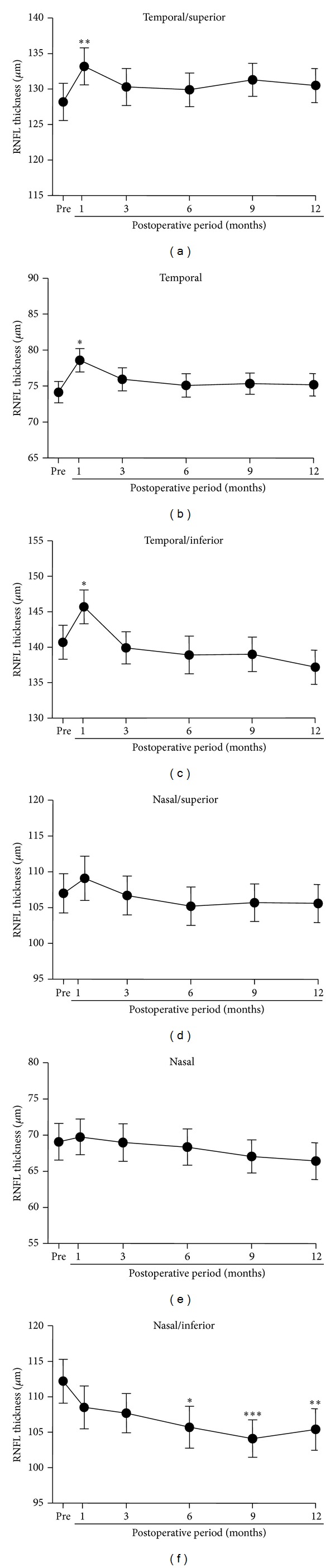
The means of the absolute RNFL thickness are plotted as a function of the postoperative periods for the superior/temporal (a), temporal (b), inferior/temporal (c), inferior/nasal (d), nasal (e), and superior/nasal (f) sector analysis. Mean ± SEM. RNFL: retinal nerve fiber layer, *P < 0.01, **P < 0.001, ***P < 0.0001.

The averages of the absolute values of the RNFL thickness obtained by sectoral analyses are plotted in Figure 5. A transient increase of the RNFL thickness was observed for all sectors (*P* < 0.01 for the temporal/inferior and *P* < 0.001 for the temporal and temporal/superior regions; Figures 5(a)–5(e)) except for the nasal/inferior sector where the RNFL thickness was progressively and significantly reduced with time (*P* < 0.001, Figure 5(f)). Multiple comparison tests demonstrated a significantly thinner RNFL at 6 (*P* < 0.01), 9 (*P* < 0.0001), and 12 months (*P* < 0.001) after surgery in the nasal/inferior sector (Figure 5(f)).

## 4. Discussion

Our results demonstrated that the RNFL thickness was significantly increased at 1 month after MH surgery but then decreased to the baseline thickness in all groups. Thus, the use of ICG, BBG, and TA did not alter the RNFL thickness significantly at 12 months following the surgery.

### 4.1. Comparison with Previous Reports Describing Toxic Effects of Vital Dyes

Visual field defects have been reported after vitrectomy using ICG [10–13]. In addition, it has been shown that the RNFL thickness measured by time-domain OCT was significantly thinner in patients with visual field defects which developed after ICG-assisted ILM peeling [12]. However in our earlier study, we demonstrated that there was no difference in the visual sensitivity obtained by standard automated perimetry among the ICG, BBG, and TA groups after MH surgery [23]. In addition, no patients complained of visual field defects. In contrast to the previous studies in which high concentrations of ICG with long exposure time were used during surgery, we used ICG at a low concentration and washed it out quickly after application which may be the reason why there was no significant decrease of the RNFL thickness in the ICG group in our patients.

Although ICG was not associated with a significant decrease in the RNFL thickness compared to BBG or TA, we found that the RGC component of the electroretinogram, the PhNR, was significantly reduced after MH surgery [23]. Ueno et al. also reported a selective reduction of the PhNR of the ERG after ICG-assisted MH surgery [29]. These findings suggest that a reduction of RGC function could develop without loss of the axons of the RGCs after MH surgery. Therefore, clinicians need to be cautious about subclinical impairments of the RGC function when ICG is used during vitrectomy.

### 4.2. RNFL Changes after Surgery

All of the patients underwent simultaneous cataract surgery. The change in the optics of the eye could possibly contribute to the increase of the RNFL thickness measured by SD-OCT after surgery [30]. However, the increase of the RNFL thickness was transient in our patients. In addition, in the nasal/inferior sector there was no increase of the RNFL thickness. Therefore, the optical change following the cataract surgery alone cannot explain the transient increase of the RNFL thickness after MH surgery. Hibi et al. have also demonstrated a transient increase of the RNFL thickness after MH surgery, and they suggested that surgical insults, such as the infusion pressure of balanced salt solution or air against retinal surface, may have played a role in an edema of the optic disc [26].

We also found that the global RNFL thickness returned to the baseline level at 6 months after the MH surgery. In the nasal/inferior sector, the RNFL thickness was significantly and progressively reduced until 12 months after surgery, indicating a loss of RGC axons. This is consistent with previous results in which the retinal thickness of the inner retina progressively decreased for several years after MH surgery [20]. Surgical insults related to MH surgery, as mentioned, may cause a loss of RGC axons.

Most recently, Balducci et al. [31] demonstrated a significant thinning of the RNFL in the temporal/superior, temporal, and temporal/inferior sectors at 6 months after BBG-assisted ILM peelings. These findings are not compatible with our results in which the RNFL thickness was not reduced in these sectors. This suggests that factors other than ILM peeling and vital dyes could produce the discrepancies between these reports. For example, the surgeon's preferred procedures, for example, location and direction of the infusion ports or infusion pressure, may be related to the loss of RGC axons.

### 4.3. Limitations of This Study

We have reported that the intraocular ICG remains in the ocular fundus for several years especially at the optic nerve head [32, 33]. This is because the intraocular ICG is taken up by RGCs and reaches the optic nerve head by axonal transport [34]. Therefore, longer observation periods over several years may produce differences in the RNFL thickness among the groups.

We have measured the RNFL thickness to represent the presence of normal functioning RGC axons. Even though there was no difference in the RNFL thickness among the groups, it does not indicate that there may not be differences in the surviving cell bodies of the RGCs. It would be interesting to compare ganglion cell complex map including RGC cell bodies [35] among the groups after MH surgery. It has been reported that the GCC thickness is reduced after macular hole surgery [36–38].

## 5. Conclusions

The RNFL thickness changed significantly only at one month after MH surgery. Sectoral analyses demonstrated a significant reduction of the RNFL thickness in the nasal/inferior region. However, the type of vital staining did not affect these changes of the RNFL thickness after MH surgery.
